# *For the Benefit of All Men*: Oceanography and Franco‐American Scientific Diplomacy in the Cold War, 1958–1970**

**DOI:** 10.1002/bewi.202000015

**Published:** 2020-11-27

**Authors:** Beatriz Martínez‐Rius

**Affiliations:** ^1^ Sorbonne Université Institut des Sciences de la Terre de Paris (ISTeP) PhD candidate Paris France

**Keywords:** Science diplomacy, oceanography, ocean sciences, Cold War, France, Franco-American relations, CNEXO

## Abstract

In the 1960s, the growing strategic importance of ocean exploration led the French government to develop greater capacity in marine scientific research, aiming to promote cooperative and diplomatic relations with the leading states in ocean exploration. Devised during Charles de Gaulle's government (1958–1969), the restructuring of French oceanography culminated, in 1967, in the establishment of the state‐led Centre National pour l'Exploitation des Océans (CNEXO). Beyond being intended to control the orientation of marine research at a national level, the CNEXO's mission was to use scientific diplomacy to balance a desire for enhancing international cooperative relations in oceanography with French ambitions to equal the USA's leading capacity to explore the oceans. Its director, the naval officer Yves la Prairie, played a crucial role in articulating scientific, national, and diplomatic interests for France in the oceans.

## 1 Introduction

In September 1955 Henri Lacombe, a French oceanographer trained in the Navy, inaugurated the first Physical Oceanography chair in France at the Musée National d'Histoire Naturelle (MNHN) delivering an opening lecture for natural scientists, professors, and students, where he lamented that:

There's no need to go very far on the study of oceanography to realize that this science has progressed thanks to foreign works. It is humiliating to realize the insignificant contribution of our country in marine research.^1^

Specialized in underwater sonar systems and physical oceanography during World War II, Lacombe was one of the few French oceanographers having frequent contact with foreign experts and projects. From his privileged vantage point, he witnessed how the US and the UK were actively exploring their underwater territories, whereas the French government was not investing enough in oceanography as it was “poorly informed about the importance of this science,” which was increasingly becoming a source of scientific prestige among the international community and an expected supplier of marine resources in a foreseeable future.^2^

Indeed, the sense of urgency conveyed by Lacombe's speech was framed in a context of growing international competition in exploring the oceans.^3^ During World War II, underwater military needs to operate and track enemy submarines shaped oceanographic research,^4^ yet in the 1950s new technological advancements gave a glimpse of the oceans’ economic potential: besides fisheries, marine environments were shown to be potential suppliers of hydrocarbons, minerals, and building materials, only accessible to those nations who could invest in exploring, controlling, and exploiting them.^5^

However, exploring the oceans presented a daunting challenge. Confronted by the oceans’ vastness and the high cost of underwater operations, international cooperation appeared to be imperative. From the late 1950s, the number of international projects to exchange data or to organize joint ventures increased, but so did the political tensions between nations. Marine research became entangled with various competing national interests: to exploit offshore resources, to control and enclose underwater territories, and to establish particular international alliances between new and emerging political blocs in pursuing geostrategic ambitions; at the same time that acquiring national technoscientific strength in oceanography was increasingly considered a source of prestige. This sparked interest in coastal states like the US, the UK, Japan, and the USSR, who directed national scientific programs towards the exploration of their underwater territory.^6^ As had already happened in other strategically valuable scientific fields, like nuclear energy and space research, during the 1960s oceanography became a key site for diplomacy, where the line between cooperation and competition blurred.

For the Cold War period, historians of science have studied the ambiguous relationships between international cooperation and competition in seeking to increase a state's international power, influence, prestige, or territorial control.^7^ Focusing on marine research, Jacob D. Hamblin has exhaustively examined how, during the 1960s, American oceanography was directed by governmental bodies, whose position combined strengthening competition with the USSR to perpetuate their leading position in ocean exploration, and fostering international cooperation to use oceanography as a tool to minimize diplomatic frictions.^8^ However, his focus doesn't address this tension for those European nations who were closely following the developments of both superpowers, and the history of French oceanography in relation with US’ strength in marine sciences remains unexplored.

In France, developing a national plan to coordinate marine research and international collaborations also appeared imperative but, as Lacombe vividly expressed, in the late 1950s France's oceanographic capabilities were far from enabling the country to participate in this arena. Oceanography was carried out with “improvised means and derisory credits,” with researchers dispersed in multiple small shore stations attached to French universities.^9^ Only a few small ill‐equipped fishing boats were available for conducting marine exploration, limiting research to coastal areas. Although there were some more advanced vessels in the fleet of the Service Hydrographique de la Marine Nationale and the Institut Scientifique et Technique des Pêches Maritimes occasionally available for academic research,^10^ the participation of, and their use by, external scientists was sporadic.^11^ French academic oceanography hardly had any international projection and collaborations in foreign projects were scarce, whereas at a national level it was disconnected from the offshore industry, which possessed advanced research technologies and more economic resources to invest in marine exploration.

It was not until 1958, when Charles de Gaulle's government entered office, that enhancing France's capabilities to explore the oceans became a matter of diplomatic concern and one of the nation's scientific priorities. Since France owns the second largest area of territorial waters across all oceans,^12^ an early investment in developing the technoscientific capabilities to access, explore, and control the natural resources it could harbor came to be considered key to position the nation as a maritime power—scientific, as well as industrial and political. The emergence of oceanography as the next valuable scientific field coincided with de Gaulle's attempt to restore France's *grandeur* with a greater national investment in technoscientific development.^13^ Given that during his tenure the French government made efforts to “keep up with the Joneses”^14^ (particularly with the US) to achieve a greater international standing,^15^ launching a coordinated national program in oceanography could efficiently situate France next to the US as pioneers in ocean exploration and exploitation, thus reinforcing France's international position. But if France aimed to become a global power in marine research, it would have to navigate through the US‐USSR antagonism, renegotiating its relationship with both superpowers through oceanography.

This study emerges from two key questions: how did cooperation and competition in oceanography come to be articulated through scientific diplomacy? And how did France's oceanographic diplomacy influence its international position during the 1960s?^16^ To answer them, I pay particular attention to the Centre National pour l'Exploitation des Océans (CNEXO), a state‐led institution created to enhance the domestic economy through the exploitation of marine resources, arguing that it was central to France's strategy of achieving a key position in relations between “the East” and “the West” in marine research. The CNEXO's director, Yves la Prairie, a former naval officer and a strong support of de Gaulle, played a crucial role in balancing the conflict between the simultaneous desire for cooperation with the US whilst maintaining the political goal of rivaling them the position as an international benchmark in oceanography.

During the 1960s, diplomatic relations between France and the US were tense because of de Gaulle's desire to challenge American hegemony in Europe.^17^ Yet from its creation the CNEXO consistently worked to strengthen ties with American oceanographic institutions who were at the forefront of marine research.^18^ Seeking to develop similar capacity and capability to explore the oceans as the Americans, the French tried to emulate their scientific administration. La Prairie went about reorganizing oceanography in France to foster bilateral exchanges and to ease diplomatic tensions.

What was the importance of having la Prairie leading France's oceanographic diplomacy and national plans? Examples from the historiography of other sciences during the Cold War show that mediators between sciences, the military, and diplomacy were most often scientists who developed the ability to merge and bridge different working worlds.^19^ Nevertheless, some studies have shown how these hybrid actors, such as la Prairie, with political backgrounds rather than scientific, played similar roles to scientific mediators, by infiltrating the scientific domain so as to influence it.^20^ As the case of la Prairie shows, for the central government to place a non‐scientist as the head of a scientific institution was not only a way to closely control the research agenda to achieve political goals, but also to regulate France's international representation. Indeed, from the moment when scientific representation at international oceanographic forums “was not anymore representing a discipline or an institution, but the nation's position,” governments needed to select reliable spokespersons who could defend the state's political positions, rather than scientific ones.^21^ For those ends, military or political officers loyal to the government appeared more appropriate than scientists to act as leaders for de Gaulle's new technocracy.^22^

Using archival sources from the National and the Diplomatic archives of France, as well as the CNEXO's archives, this research charts the establishment of this institution as a center for French science diplomacy. From the initial moves within France to transform oceanography by first establishing a new scientific council, and to later centralize research efforts in the CNEXO, scientific diplomacy grew to become a key component of the institution's work. Eventually, in 1970, France signed a bilateral cooperative agreement with the US, in which both countries attempted to sideline political disagreements and displayed a will to cooperate in oceanography, defining their joint goal as being “to advance [the] study and effective utilization of the sea *for the benefit of all men*.”^23^ Although the sentence stressed the importance of sharing the knowledge produced from their joint projects, it concealed the dominant position both countries were adopting in their desire to explore the oceans and increasingly exploit its riches, thereby embedding a constant tension between competition and cooperation.

## 2 An Initial Attempt to Boost French Oceanography: The Comité “Exploitation des Océans” (1958–1965)

That France was lagging behind in oceanographic exploration was evident to those few French oceanographers who had participated in international meetings and projects during the late 1950s. In 1960, Lacombe and a dozen French oceanographers and experts were charged by the Délégation Générale à la Recherche Scientifique et Technique (DGRST) to write a report addressed to the Prime Minister's cabinet, urging the government to foster national investment in oceanography.^24^

The report detailed the oceanographer's concerns regarding France's lack of capabilities to meet the growing international effort to explore the global ocean. The International Geophysical Year (IGY, 1958–1959), during which Lacombe had participated in cooperative surveys to study the Mediterranean‐Atlantic water exchange, proved the effectiveness of promoting international coordination for exploring the oceans.^25^ Resulting from the IGY, the Special Committee on Oceanic Research (SCOR)^26^ planned the International Indian Ocean Exploration (IIOE) for 1962–1963 where, according to French experts, France had to participate for the “strategic, political and human interests” that the country had in that area.^27^ At the same time, UNESCO created the International Advisory Committee on Marine Sciences (IACOMS), a consultative council formed by nine representatives of member nations in which France was going to have a seat, to develop an international program of ocean research and expert training.^28^

However, the strongest pressure to invest in oceanography came from NATO. In a meeting of its Science Committee in April 1959, the Science Adviser and chairman Norman F. Ramsay^29^ emphasized that “The North Atlantic is so central to NATO that it is the identifying feature in the name of the organization. Nevertheless, the largest oceanographic research vessel at present studying the North Atlantic belongs to Russia.”^30^ American anxieties regarding their control of the oceans grew stronger after realizing that the Soviet Union could be gaining an advantage in charting the seas, as evidenced in a secret report issued by the British Navy entitled “Oceanography and Defence in the USSR, 1956–1958.”^31^ NATO's Science Committee members agreed that an exhaustive survey of the oceans could only be efficiently and rapidly pursued through cooperative projects in military oceanography among its allies, decision that crystalized in the creation of a NATO Subcommittee on Oceanographic Research (ORC) in September 1959.^32^

The ORC was established by prestigious oceanographers from the allied countries, being France's representatives Henri Lacombe and Jean‐Marc Eyrès, director of the French Naval Hydrographic Service. The committee's mission was to organize a coordinated program to answer NATO's underwater surveillance needs in submarine operations, anti‐submarine warfare, meteorology, marine transportation, and in understanding the effects of radioactive fallout. However, an initial analysis of oceanography in the allied countries demonstrated their main weakness: the scarcity of competent, skilled researchers and technicians, who possessed the expertise required to undertake complex marine studies especially in physical oceanography.^33^ ORC's priority became, thus, to encourage the allied countries to foster the growth of an oceanographic labor force, to facilitate the exchange of researchers, technologies and ideas, and to build oceanographic vessels available to support NATO's joint surveys.^34^

NATO's call for oceanographers reached the French government at the time when France's position in the Alliance was wavering. De Gaulle had been arguing for a tripartite leadership of NATO between the US, Great Britain and France, a position that would grant him power in the decision‐making about nuclear weapons’ management. For the General, demonstrating France's technoscientific strength was essential to appeal for a powerful seat in NATO, but for oceanography the 1960 report by French oceanographers addressed to the Prime Minister's cabinet specified a concern about France's lack of oceanographic capacity and its impact on their ability to wield any power in NATO's ORC. Its authors insisted that French participation in NATO's oceanographic projects “must be on the same level as that which our country occupies in the Alliance” and they warned that, if France's lack of vessels, funding, resources and personnel persisted, the country could find itself in a humiliating position in the international arena.^35^

As framed in the fourth Development Plan (1960–1965), the government's answer materialized in the Comité Scientifique “Exploitation des Océans” (COMEXO), a committee formed by eight experts handpicked by the Ministry of Research in charge of remediating France's hitherto lack of attention to marine exploitation. The group was formed by the heads of the main laboratories in marine sciences, including Lacombe and Eyrès as the links with NATO's ORC.^36^ From 1960 to 1965, the DGRST granted the COMEXO 14 million dollars,^37^ which were invested in supporting forty research grants in fundamental research per year and in participating in international projects under the leadership of NATO and UNESCO.^38^ However, without a previous plan to organize the allocation of funding, the economic support was disseminated among the laboratories that requested it—which were, in most of the cases, those directed by the COMEXO's members.

The largest allocation of funds went to research in the Mediterranean Sea. Between 1961 and 1965, twenty projects were supported there for fundamental research and for recognizing its hydrology and seabed topography, whereas only nine were devoted to the Atlantic. On the one hand this disparity was simply because most of the French research centers were located along the Mediterranean coast, however there were also specific geopolitical reasons for the larger interest in this sea. Since the early 1960s, the Mediterranean had become the theater of NATO's underwater operations, being even called “an American lake” by the American press.^39^ NATO military officers’ fear that Soviet submarines could move in and out the Mediterranean through the Gibraltar strait without being detected promoted many studies on physical oceanography in this area through NATO's newly created Supreme Allied Commander Atlantic (SACLANT) Anti‐Submarine Warfare (ASW) Research Center located at the Italian village of La Spezia.^40^ The COMEXO facilitated collaborations between French and American oceanographers in exhaustively charting the Western Mediterranean: The well‐known commander Jacques‐Yves Cousteau and his team of technicians furnished their vessel *Calypso* equipped with new sounding technologies, while Lacombe led some studies on seawater circulation through the Gibraltar strait.^41^

In 1962, despite the COMEXO's efforts, French oceanography was still lagging, as a headline in the British journal *The New Scientist* stated.^42^ When the COMEXO's activities finished at end of the fourth Development Plan, in 1965, the Committee's biggest achievement had been the construction of the *Jean Charcot,* the first oceanographic vessel in France able to carry out oceanographic surveys on the high seas. However, its members acknowledged that they had not been able to establish an effective training plan, and research remained distributed across more than hundred laboratories.^43^ To resume their activity, the COMEXO appealed for the government to transform the committee into a stable and autonomous institution, free to manage its budget without the DGRST's supervision, with decision‐making capacity about projects, training, and France's international representation.^44^

The COMEXO's request was heard by the government, but the restructuring was far from the oceanographers’ desires. The French Council of Ministers decided to move the decision‐making about oceanography's organization from scientific committees to the hands of policy‐makers close to the government, with the goal of aligning the orientation of oceanographic research with the state's pursuit of industrial, technological, and economic self‐sufficiency.

## 3 Contesting the American Hegemony in Oceanography: The Birth of the CNEXO

During the early 1960s, Franco‐American diplomatic relations were deteriorating. In 1964, Charles de Gaulle withdrew the French Mediterranean fleet from NATO's command after suspecting that the American and Italian governments were secretly supporting Algerian independence.^45^ Disagreements about nuclear deterrence further strained Franco‐American relations until 7 March 1966, when de Gaulle withdrew from NATO's Allied Command, announced that France would not join NATO's nuclear planning group, and refused to maintain NATO's headquarters in Paris. In moving away from the US, in an act of rapprochement de Gaulle now looked to develop more cordial relations with the USSR intending to create a “Europe from the Atlantic to the Urals.”^46^ France, according to the general, could come to play a pivotal role in overcoming the Cold War dichotomic division, thereby reinforcing its international prestige and power.

For the American government, oceanography was increasingly considered a valuable field to boost the economy. President Lyndon B. Johnson, in office from 1963 to 1969, was excited about the future possibilities that marine resources could offer to his country and did not hesitate to devote huge national efforts to promote oceanography.^47^ In 1965, the US Congress designed a national plan to orient oceanographic research towards the fulfillment of national needs, which crystallized in the creation of the Commission on Marine Science, Engineering and Resources.^48^ Its leader Edward Wenk Jr., a naval engineer expert in submarines, had been playing diverse policy advisory roles at the White House under the administrations of Presidents Kennedy and Johnson before being put in charge of it.^49^ According to the French press, the American investment in the Commission (more than 200 million dollars for 1967) represented an unprecedented national effort devoted to oceanography, only comparable to the creation of NASA nine years before.^50^

In France, the government was closely following these actions, fearing that the US was “preparing to launch an offensive to discover all the richness of the seas.”^51^ To avoid accumulating a “regrettable delay” in a domain with increasing political, economic, and military importance, they decided to create a structure equivalent to the emerging one in the US by placing the exploitation of the oceans as one of the nation's critical scientific domains to advance for its “scientific importance and its vast potential for economic boost.”^52^ The subject of interest was not oceanography nor exploration, but exploitation: Their focus was on developing scientific, technological, industrial and territorial knowledge to achieve an effective exploitation of oceanic natural resources—notably oil, gas, and manganese nodules, but also for exploiting fisheries more efficiently.

The Centre National pour l'Exploitation des Océans (CNEXO) resulted from that desire. Through this institution, the French government aimed to closely control and lead international relations in oceanography, as well as to concentrate efforts in improving the nation's capacity to exploit offshore resources—which would contribute to increase France's *grandeur*.^53^ The military potential of fostering oceanography was not specified as a priority, but neither was it disregarded. Oceanographic research projects of direct military interest would continue at the navy's Hydrographical Service, supported with the budget devoted to the military, even though collaborations with the CNEXO were frequent in the following years.

The new‐born CNEXO was not a regular scientific laboratory, but an institution strongly controlled by the government. The orientation of its programs was going to be decided by experts from the Ministries of Research, Navy, National Education, and Finances, as well as representatives from the oil industry and fisheries, who would gather in an Administrative Council.^54^ The General Director of the CNEXO would participate in their meetings, bridging the government, the Administrative Council, and the scientific community. Given the importance of selecting an appropriate mediator, Alain Peyrefitte, the Minister of Information of de Gaulle's administration, handpicked a candidate: Yves la Prairie.

Trained as a naval officer, la Prairie fought in the French Navy during World War II and became a member of the French Résistance. After the war, his military career took him to the Middle East and North Africa, where he gained experience in international relations. In 1954, after settling in Paris, he obtained a position at the Commissariat de l’Énergie Atomique (CEA) as the secretary of Jacques Yvon, director of the department of Atomic Piles, where he met policy‐makers, scientists, and members of the Ministries’ cabinets, until he was promoted to be the Technical Advisor to Gaston Palewski, Minister of Scientific Research, from 1962 to 1965. According to la Prairie's memoirs, Peyrefitte was looking for a director who was:

Not an admiral, nor a Naval engineer, but someone younger and external to the Military […]. Neither university scientists, given the CNEXO's orientation towards applied and economic goals […]. Nor experts from the private industry, at least at the beginning.^55^

La Prairie was deemed the right person, whose complete lack of oceanographic knowledge was irrelevant: More important was his civil service background, mixed enough to mediate in political, military, scientific, and international grounds. At the CEA, la Prairie had mingled with all kind of experts, from the Government to the laboratories, had developed firsthand knowledge about how big science institutions worked, and knew how government policies were articulated at all levels until transformed into scientific projects.^56^ Moreover, besides his experience, la Prairie considered himself sincerely Gaullist, something well‐known among his fellows at the Ministries.^57^ That loyalty was fundamental during the Cold War's most tense period, when in some cases Western governments had distrusted or even dismissed the heads of research centers for being close to communism.^58^

In the new institution, scientists were relegated to the role of technical advisors to the CNEXO's director at the Scientific and Technical Committee. Although most of its members, handpicked by la Prairie, had been part of the former COMEXO, the new CNEXO differed from the previous committee in that it would demote their decision‐making capacity under the director, transforming oceanography's management into a state‐led structure re‐orienting scientific research at all levels, from higher education to public laboratories, towards an ocean economy.

The CNEXO became operational in April 1967, establishing its headquarters in a CEA office in Paris. La Prairie recruited his team of experts, hand‐picked to guarantee their loyalty, from among his fellows in the CEA and the military. Marine technologies under COMEXO's control were transferred to the CNEXO: Three oceanographic vessels (the *Jean Charcot* among those) and three more under construction, diving technologies (the submersible *SP‐3000*, the deep‐sea submersible *Argyronète* and the bathyscaph *Archimède*), the fixed offshore platform for scientific research *BOHRA I*, and the contracts previously granted by the COMEXO.^59^

The CNEXO was first used as a tool for diplomacy with the USSR.^60^ Although Franco‐Soviet relations had been tense during the first years of the decade, de Gaulle sought an accord with the USSR to develop economic relations that would benefit both countries. In June 1966, three months after quitting NATO's Allied Command, de Gaulle was warmly received in Moscow, where both nations agreed on strengthening diplomatic ties through trade and scientific cooperation.^61^ Among the fields deemed relevant, they envisioned a bilateral cooperation in oceanography, which materialized in a cooperative treaty signed one year later, just after the CNEXO was created.

In that treaty, both countries agreed on pursuing joint research projects in marine biology and fisheries, geology, geophysics, and deep‐sea diving technologies;^62^ but its terms, decided by the Ministry of Research, were criticized by CNEXO's Scientific and Technical Committee. Two of its members, Lacombe and Jacques‐Yves Cousteau—the famous naval officer, explorer, and filmmaker—were concerned that the Soviet strategy was to use the French research technologies and resources to advance their competition against the US in marine exploration. According to Cousteau's team,

the rush of the Soviets is clearly explained by their eagerness to reach the US, being also some years behind France. Because of this delay, the Soviets can obviously only provide the Cousteau Group with a scientific contribution which, without being negligible, is however secondary.^63^

They complained that the envisaged cooperation was unequal, since France would invest much more than it what was going to receive. Although la Prairie agreed that the program was far from the CNEXO's interests and budget, the government decided to support the French‐Soviet cooperation to maintain cordial diplomatic relations.

For the next two years, French and Soviet scientific parties exchanged visits to define the terms of their cooperation and build mutual confidence. French experts visited the USSR seven times, touring laboratories and Soviet research facilities, whereas the Soviets only visited France twice. However, by mid‐1968, the terms of the cooperation were still not clear. Disagreements, related to the research methods both at sea and in the laboratory, persisted, and for some projects—especially the ones related with technological development and exchanges—they couldn't agree on the goals to pursue.^64^ Joint researches in fisheries, marine geology, and geophysics began in late 1968, but the CNEXO's direction was going to pay notably less attention to those rather than to joint projects and exchanges with the Americans.

## 4 Between Competition and Cooperation: Shaping the CNEXO in the Mold of American Oceanography

When the CNEXO's activities began, Franco‐American diplomatic relations were at their lowest point. This period is considered as the peak of de Gaulle's illusion of independence when, after withdrawing from NATO's Allied Command, he sought to promote national independence by moving closer to the Warsaw Pact countries and the USSR, while constantly disagreeing on the decisions taken by the US on nuclear strategy, the Middle East, and the Vietnam War.^65^

By signing a cooperative agreement with the USSR, the CNEXO had proved its utility as a tool for scientific diplomacy, but there were still tensions to ease with the US. Despite the strained diplomatic relations between both countries, la Prairie initiated some exchanges with American experts in the pursuit of friendly relations. However, by establishing ties with American institutions, la Prairie and his team had another goal: to gather information about American organization, structure, centers and style of research, in order to implement similar measures at the CNEXO. The CNEXO, hence, became a mechanism of scientific intelligence, besides an intermediary for scientific collaborations.^66^

Soon after the CNEXO's establishment, la Prairie initiated a correspondence with Edward Wenk Jr., Executive Secretary of the National Commission on Marine Science, Engineering and Resources. Created in June 1966, the group was commissioned to implement a planning and coordination policy of the US’ oceanic research, and the CNEXO had awaken Wenk's interest in the French coordinating plan.^67^ In his letters, la Prairie insisted him on how similar the CNEXO's and the Commission's concerns and interests were, anticipating in a private letter to the Minister of Research a likely future cooperation with the US.^68^ Besides Wenk's correspondence exchanging their points of view, la Prairie obtained a second source of information through the French Embassy in Washington, requiring them to report him about American advances in oceanography.^69^ Those reports included discussions held in the US Congress, the annual budget devoted to oceanography compared with other countries’ investments, and details on their higher education system. Based on those reports, CNEXO experts came to consider that the successful structure for supporting research in the US relied on the tight articulation between scientific research and the industry, the investment in fundamental science, and the American oceanographic research centers.^70^ From those sources of information, la Prairie promoted a restructuring of French oceanographic research.

The training of experts and their absence in international forums was still the major problem. To implement solving measures, la Prairie relied on his right‐hand scientist at the CNEXO, Xavier le Pichon, a young marine geophysicists who had spent six years in the prestigious research center Columbia University's Lamont‐Doherty Earth Observatory (LDOE) in New York.^71^ By comparing the French training structure with the American one, le Pichon advised that the CNEXO must recruit the best young researchers from universities and encourage them to travel between research centers in France and abroad.^72^ On the other hand, although the US largely supported fundamental research, frequently addressing military objectives, the training of their oceanographers was increasingly being linked to the industries devoted to marine resources exploitation, where trainees had to spend part of their formation.^73^ Inspired by this training system, the CNEXO offered 30 contracts per year for enhancing PhD instruction at national companies and abroad. Those grants required students to work at CNEXO's joint industrial laboratories, where they would develop skills in that sector; while continuing with their fundamental research projects at national or foreign universities. This decision constituted a complete transformation of what was deemed to be a scientific career in oceanography. Whereas the former COMEXO supported training in fundamental research under the leadership of a single university patron to engage young oceanographers in a future oriented towards academia, the CNEXO's industrial training and mobility opened two future paths for researchers, in which industrial and academic research were entangled.^74^ These two measures were directed at creating a pool of experts, capable of working in international, cooperative and multidisciplinary projects, and skilled in using cutting‐edge technologies. By visiting foreign research centers, they would learn other competences beyond scientific skills: the capacity to effectively represent France abroad, to report on their return about developments in foreign countries, and to evaluate how French potential was considered in the international community, which was essential to the CNEXO's scientific diplomacy. ^75^

This pool of young researchers soon became a new valuable source of information for la Prairie—and thus, for the French government—through those who undertook training placements in the US with the CNEXO's grants.^76^ PhD researchers in marine geophysics, such as Jean Bonnin and Bruno Leclerc du Sablon, were among the first to travel to the LDEO under the CNEXO's sponsorship in 1968. After their one‐year stay in the US, they exhaustively reported to la Prairie about the skills they had learned, the technologies they had brought, the theoretical framework in which American researchers worked, the projects supported by the American government, and the scientists they had met.^77^ These reports demonstrate one of the roles that the CNEXO's labor force was going to play in the years to come: They were, consciously or not, a source of scientific intelligence, which imported to France information, expertise, and the American model of research as tools to boost French oceanography.

The CNEXO's measures to control the national budget devoted to marine sciences and technologies, its focus on applied research, and the concentration of efforts in exploring the Atlantic, did not satisfy all French researchers. Many feared that they would lose their freedom and funding if their projects were not adjusted to the national plan. Since the CNEXO barely supported fundamental research, Lacombe and other members of the Scientific and Technical Committee pushed to find a mechanism in which fundamental research could be supported in universities. Disagreements reached the Ministries, where the Conseil National de la Recherche Scientifique (CNRS) decided to create its own Oceanographic Commission to manage the budget for marine sciences at universities and public laboratories.^78^ Lacombe was part of the CNRS committee, mediating between the CNEXO and the CNRS. This system allowed for the maintenance of research autonomy at universities, albeit with fewer resources and less funding than at the CNEXO. Eventually, even la Prairie agreed on the need to support a core of fundamental research within universities, which was essential to provide basic training for his new team. In turn, the CNEXO had absolute control over French research vessels and technologies like submersibles, which empowered the institution to decide in which projects French resources were to be used.^79^

Regardless of the opposition of some French researchers, for many the CNEXO could provide them with cutting‐edge equipment and the opportunity to be part of international projects if they were willing to adapt their research to the CNEXO's goals.^80^ For that reason, during the two initial years of the CNEXO, teams at universities and laboratories at Brest, Paris, Grenoble, Nantes, Rennes, Marseille, Bordeaux, Caen and Montpellier asked for the CNEXO's support, which resulted in almost 40 contracts to chart and explore the Atlantic and the Mediterranean continental shelves, marine biology and geochemistry to improve the exploitation of fisheries, and technological developments.^81^

## 5 La Prairie as a Diplomat: International Representation and Scientific Diplomacy

As the deep oceans emerged as new fields for diplomacy, the number and frequency of political congresses and meetings devoted to the topic steadily increased. The creation of the CNEXO responded to the French government's need to coordinate their representation within the international arena.

In his role as General Director of the main French oceanographic institution, la Prairie had the right to act as, or to nominate from among the CNEXO members, the French representative at all international meetings, either scientific congresses or diplomatic encounters. During the early years of the institution, he occupied decision‐making positions at the UN Expert Meetings in Marine Science and Technology, UNESCO and its International Oceanographic Commission (COI), and the Commission Internationale pour l'Exploration Scientifique de la Méditerranée (CIESM). La Prairie assured a smooth articulation between international meetings, marine research and the French government, informing the concerned Ministries through letters, reports, and personal meetings about the movements, decisions, and negotiations held during those congresses.^82^

But la Prairie's role was not merely channeling information to the government. He acted as a diplomat, announcing the huge French projects and investments in exploring the oceans among the international community, and driving negotiations intended to establish cooperative relations. From 1967 to 1969, several states interested in the French reorientation of scientific research—including Sweden, the UK, Mexico, Israel, Japan, Cuba, and Portugal—approached la Prairie seeking his advice to create similar coordination structures. La Prairie visited those countries to assure the establishment of strong diplomatic relations through oceanography, which materialized in joint projects and frequent exchanges of scientists during the following decade.

Oceanography contributed to France's reconciliation with the US. In March 1968, la Prairie traveled there for the first time on an official trip to participate in the second meeting of UN experts in Marine Science and Technology in New York. His visit coincided with a major event in American oceanography: On 8 March, President Johnson announced the International Decade of Ocean Exploration (IDOE) from 1971 to 1980, planned by the Council led by Edward Wenk Jr.^83^ The effort, to which the US Government was going to devote more than $200 million, constituted a major initiative to promote a cooperative exploration of the oceans as a means to foster, according to Johnson, “a stable and international peace.”^84^

La Prairie used his trip to meet prominent experts—both scientists and policy‐makers—of American oceanography. Wenk conducted private tours for him of the main oceanographic centers^85^ and organized la Prairie's visit to the White House to meet the American vice‐president Hubert H. Humphrey, who was overseeing American ocean policy under Johnson's administration. Despite diplomatic tensions between both countries, Humphrey warmly welcomed la Prairie, exclaiming that he was and would always be “deeply Francophile.”^86^ They discussed how their institutions, the future National Oceanographic and Atmospheric Administration (NOAA) and CNEXO,^87^ could further coordinate oceanography, envisioning a Franco‐American cooperation through them. The recently announced IDOE was on everyone's mind, infusing urgency to establish fruitful relations to prepare the oceans exploration venture that was going to start in 1970.

But besides establishing friendly relations, la Prairie's activities in the US had a second goal. From his visit to American centers, as well as from reports facilitated by the French embassy, the CNEXO held large amounts of information about the American research institutions, from detailed maps of their organization to statistics about the centers’ capacities and space devoted to each activity.^88^ La Prairie's team came to identify the high‐quality of the US research centers, which were big, equipped with heavy research equipment, and a structure that facilitated cross‐disciplinary studies, as the keys to American oceanography's success. Inspired by the information gathered, the CNEXO planned its own: the Centre Océanographique de Brest (COB).^89^ Situated on the Atlantic shore, the government aimed to boost the exploration of its continental shelf, a submerged territory equal to one third of France's emerged lands.^90^ The COB would control the country's research resources: from vessels and research technologies to a critical mass of experts, who were to be hired from universities around the country.^91^ As stated in an article published in *Science,* the COB was “both a symbol and the first fruits of a new concentrated national attack on oceanographic problems,” concluding that “[France] seems to feel that in oceanography an investment of this size will keep them in a competitive position with regard to other nations.”^92^

## 6 Enhancing Franco‐American Relations through Oceanography: The Bilateral Agreement of Cooperation

Meanwhile, de Gaulle's national and international prestige declined. In the international arena, the General's support of Arab countries during the Six‐Day War (1967) while imposing embargoes on Israel was not well received among the western allies, particularly in the United States. In focusing on restoring France's international prestige, the General was judged to have overlooked various national problems. The French population's discontent exploded in May 1968, when a student revolt transformed into a national uprising against de Gaulle's policies. These events showed the General the limits of his politics of *grandeur* and forced him to resign from the presidency in April 1969, after witnessing how much his public support had decreased.^93^

Before his resignation, de Gaulle's government overlapped three months with the new American president Richard Nixon, inaugurated in January 1969, which constituted the height of diplomatic rapprochement from both sides. The reasons for this were two‐fold: On the one hand, Nixon and de Gaulle shared a mutual esteem, which resulted from a similar political ideology; and on the other, Nixon's quest for reconciliation with France reflected his decision to focus on enhancing the national economic situation instead of investing in efforts to shape Western Europe's politics and economy.^94^

In February 1969, de Gaulle received President Nixon in Paris. To strengthen diplomatic ties, both countries agreed to cooperate in strategic scientific domains: Nuclear energy, space research and oceanography were selected; as well as medicine, transportation, protection of the environment, scientific information, and agronomy. Although nuclear and space research had been subjects of Franco‐American cooperation since the end of World War II, only in oceanography had a memorandum of understanding been drafted. After one year discussing its terms, in February 1970, la Prairie and Wenk, as the Executive Secretary of the Marine Science Council, signed a treaty of bilateral cooperation.^95^

Only three years after the CNEXO's creation, France could rely upon its new strength in oceanography to pursue closer diplomatic relations with the Americans. But what constituted that power? In 1970, the institution controlled six oceanographic vessels and three manned submersibles, but those were the same resources existent at the end of the COMEXO's period. The COB was still under construction, and would not host its own team of researchers until 1971. Problems in coordinating the budget among different ministries remained, as well as frictions with the CNRS and the Ministry of Higher Education. Whereas French oceanographers numbered 450, the US had by then around 5,800; and while the CNEXO was supporting 40 PhD students per year, the US was annually producing 510.^96^ However, the national budget devoted to the CNEXO had tripled: from $4 million in 1967 to $12.8 million in 1970,^97^ and the institution was well‐represented in international forums of marine exploration.

Most importantly, by 1970 France was the only country that had created a coordinating structure equivalent to the American one. Wenk and la Prairie agreed that both countries shared interests and concerns relating to the oceans, and they proudly affirmed that they were “one step ahead in the ocean race.”^98^ Hence, the American interest in signing a treaty of bi‐lateral cooperation with France did not rely on French capabilities to explore the oceans, which were much smaller than theirs, or on the basis of past scientific achievements, which were scarce, but on the state's structure of scientific coordination, the CNEXO, itself. The plan to reorganize French marine research and boost technological development unifying them under a single state‐controlled institution facilitated these new diplomatic relations, through which France renegotiated its position in relation to the US.

The Franco‐American treaty relied on three agreement points. Firstly, any cooperative project should, above all, “advance study and effective utilization of the sea for the benefit of all men.” Secondly, it should “strengthen and support multilateral oceanographic programs under the aegis of the IOC and other international agencies,” and thirdly it should “foster acceleration of national programs in each country by building on the research and experience of the other.”^99^ The discourse of supporting science bilaterally for a common good was not exceptional, but rather something common. In the context of the Vietnam War and the increasing discredit of the US, the American administration didn't want another race against the USSR. Instead of phrasing their intentions through a mutual national interest, policy‐makers and oceanographers transformed their rhetoric to praise the use of international cooperation to promote goodwill “for the benefit of all mankind.”^100^

For Franco‐American diplomacy, prioritizing cooperation reaffirmed the diplomatic will of maintaining friendlier relations, following the Nixon administration's interest in improving relations with France. However, conversely France insisted on referring to ocean exploration as a *race*. Jacques Perrot, a former military officer who became la Prairie's right‐hand at the CNEXO, synthesized the on‐going competition by asserting, during a conference at the Association of National Naval Reserve Officers, that:

[The oceanographic adventure] is not a 100‐meters race […] but a long‐distance race, where tactics are as important as breath. […] From the very beginning, we need to take the leadership; once the race has begun, the efforts to leave behind the bulk of racers and find a new place [at the forefront] will be excessive if compared with the efforts required at the beginning [of the race].^101^

In this framework, the Franco‐American agreement can be reinterpreted: It reflected the position adopted by both countries at the forefront of ocean exploration, recognizing themselves as its leaders. “The benefit of all men,” or the outcomes from their cooperation, would be defined by the needs of those two states, who would decide where to invest, for what purpose, and in cooperation with whom.^102^

Just after the treaty was signed, it materialized in their first cooperative project, FAMOUS (French‐American Mid‐Ocean Undersea Study).^103^ The study was located in international waters, over the mid‐Atlantic ridge (700 kilometers west of the Azores). To demonstrate their joint strength to the international community, the expedition relied on manned submersibles from both countries that could plunge to areas inaccessible to other states. Diving 2,500 meters deep, they reached for the first time the underwater spreading edge of two tectonic plates, the Eurasian and the North American. The project not only contributed to fundamental research, but it also staked out France's position as one of the main technological oceanographic leaders.

## 7 Conclusion

After retiring, la Prairie recognized the essence of the role he played at the CNEXO by declaring that “I have been a living and permanent link between politics and science: the scientist's interpreter for the political world, an interpreter of politics for the world of science.”^104^ But as this paper has shown, beyond being merely an interpreter, la Prairie was an essential piece of the government's strategy to take control of oceanography's orientation. He led the restructuring of marine research at a national level and embodied France's interests in the international arena. Even though the selection of la Prairie as the CNEXO's director might be considered a contingent event inside the process of developing a national plan in oceanography, choosing the right mediator was of the utmost important for aligning scientific, national, and diplomatic interests. As the initial case of the COMEXO demonstrates, scientists’ interests and their style of managing research often conflicted with the state's goals. Selecting a mediator who was not a scientist proved an effective way of maintaining the government's goals as the main priority, both at national and international levels.

France's oceanographic diplomacy relied on the CNEXO and on Yves la Prairie. The CNEXO's creation mirrored France's intentions to reinforce its economy and emerge as a global power by extending its capability in exploring the oceans. As I have argued, to do so it needed to mobilize a strategy that balanced competition and cooperation with other nations, particularly the US and the USSR. France's oscillation between both positions was defined by the situation of French international relations: When the political environment was conductive to rapprochement, the CNEXO mediation worked to ease diplomatic tensions by promoting cooperative relations. However, la Prairie's eagerness to collect information from American oceanography through a wide range of sources—the French embassy, scientists, and himself—evidences the permanent context of competition. In the ocean race's framework, la Prairie's team imported, evaluated, and implemented within the French oceanographic structure the American elements considered most desirable to boost marine research, from the organization at NOAA to the research center's arrangement at the LDEO.

Eventually, the ambitious goal of controlling France's marine research was not completely fulfilled. La Prairie's decisions conflicted with the oceanographic community, who perceived that their budget and freedom of research was potentially to be subordinated to the CNEXO. After la Prairie's retirement, the CNEXO remained the main oceanographic center in France and it was still charged with coordinating French representation in international programs and committees. However, it progressively became a center devoted to applied research, which offered its services to public and private organizations, whereas fundamental research—that is, research lines not directly oriented to industrial or military needs—support was maintained by the CNRS at universities and public laboratories.

## Footnotes

1 Lacombe 1956. All direct quotations originally in French have been translated by the author.

2 COMEXO, “Rapport du Comité d’études ‘Exploitation des Océans,’” 12 March 1960, p. 11, Paris, Archives Diplomatiques de France (hereinafter ADF), Collection: Relations Culturelles, Scientifiques et Techniques (hereinafter RCST), 236QO, Box 35.

3 The rhetoric of *ocean race* or *competition* was ubiquitous in French and American political speeches during the 1960s and 1970s. The American rhetoric of *race* is discussed in: Adler 2019, on 101–134 (Chapter: “Cold War Science on the Seafloor”); Hamblin 2005, on 140–176; also Robinson (forthcoming).

4 About the relations established between military patronage and oceanography, see: Hamblin 2005; Oreskes 2003; Rainger 2000b; Weir 2001. For analysis about military patronage outside the US, see: Robinson 2018 for the UK; Camprubí and Robinson 2016 for Spanish‐British relations.

5 New technologies included sonar, marine geophysical devices and offshore drilling technologies. About the impact of new technologies on the enhancement of scientific knowledge about the oceans, see Rozwadowski and Van Keuren 2004. The relation between oceanography and the exploitation of marine resources has received notably less attention than the military patronage, exceptions are: Adler 2019, on 101–134 (Chapter: “Cold War Science on the Seafloor”); Dorsey 2016; Rozwadowski 2019; Rozwadowski and Van Keuren 2004.

6 Hamblin 2005 has analyzed the American national programs, whereas Robinson 2018 has studied the creation of the British National Institute of Oceanography to articulate British military interests with oceanography in the shape of a national program.

7 For France the tensions between science competition and exchanges with the US to reinforce France's prestige after WWII have been explored by Hecht 1998 (nuclear research) and McDougall 1985 (space research), whereas Krige 2014 has studied the inherent tensions embedded in exchanging and adopting foreign technologies, methodologies, and research values in transnational space research and rocketry between France and the US. For the earth sciences, Adamson 2016 has explored Franco‐American diplomacy through uranium research in Morocco, between scientific intelligence and exchanges.

8 Hamblin 2005.

9 COMEXO, “Rapport du Comité d’études ‘Exploitation des Océans,’”12 March 1960, p. 11, ADF, RCST, 236QO, Box 35; COMEXO, “Rapport d'activité annuel du Comité scientifique ‘Exploitation des Océans,’” 16 March 1967, p. 1, Paris, Archives Nationales de France (hereinafter ANF), Collection: Marine Marchande, Centre National pour l'Exploitation des Océans (hereinafter MM CNEXO), 20060160, Box 1.

10 Throughout this paper, I use the term “academic” to differentiate scientific research conducted at universities and laboratories supported by the Conseil National de la Recherche Scientifique (CNRS), from that conducted inside, or in relation with, the military or the offshore industry, which embraces private and public companies devoted to oil and gas exploitation, fisheries, and marine technology development.

11 COMEXO, “Rapport d'activité annuel du Comité scientifique ‘Exploitation des Océans,’” 12 March 1960, p. 3–4, ADF, RCST, 236QO, Box 35.

12 As a matter of comparison, France's Economic Exclusive Zone (EEZ, term coined after the 1982 United Nations Convention on the Law of the Sea to border national territorial waters, where nations have exclusive rights to explore and exploit natural resources) is the second largest in the world (10.7M km^2^), only surpassed by the US’ EEZ (12.16 M km^2^).

13 About de Gaulle's re‐organization of scientific policies, to transform technosciences in a pillar of the nation's development, see: Jacq 1995; Jacq 2002, Lelong 1999; Simoncini 2018.

14 English idiom referred to someone who constantly compares his or her goods to his/her neighbors’, as those are considered as a benchmark for social or economic status.

15 Robinson, on 187 discusses how the British investment in fundamental oceanographic research was justified as “keeping up with the international Joneses.”

16 Scientific diplomacy is here understood as a national mechanism or plan to influence on diplomatic relations through scientific research, cooperation, or exchanges. For a detailed account on scientific diplomacy, see Ruffini 2017.

17 About France's international position and particularly Franco‐American diplomacy during de Gaulle's tenure, see: Martin 2013; Reyn 2010, on 307–354 (Chapter: “Grand Designs Go Bankrupt: From Divergence to Accommodation, 1967–1969”).

18 For a fundamental study about the American quest for hegemony in Europe, see Krige 2008. Hamblin 2005 describes the primacy of American research institutions.

19 A foundational work on scientists as intersectional actors is Doel 1997. Turchetti et al. 2012 have discussed the role played by “flexible identities” between scientist‐diplomat‐politician in hybrid domains. About American oceanographers’ mediating role with the military and the state government, see: Mukerji 2016; Rainger 2000a; Rainger 2000b; Weir 2001; Hamblin 2005; and in other countries, see: Robinson 2018; Roberts 2013. About scientific mediators in earth sciences, see Turchetti et al. 2014. The concept *working worlds* was coined by John Agar to describe “arenas of human projects that generate problems.” Agar 2012, on 3–6. Here I use it to highlight the different skills that these hybrid figures needed to possess to navigate a range of working cultures: the sciences, politics, diplomacy, the military, and the industry.

20 Although less commonly found in scholarly literature, some examples can be found at: Hamblin 2005; Turchetti 2019. About how Lyndon B. Johnson's administration officials promoted environmental sciences through a scientific diplomacy, see Doel and Harper 2006. Krige and Barth 2006 have defended the relevance of exploring the role played by diplomats in scientific diplomacy.

21 Hamblin 2005, on 169–170.

22 The creation of a French technocratic government after World War II was strongly criticized by intellectuals and scientists; Hecht 1998.

23 Edward Wenk Jr., “Terms of Reference for Marine Science Cooperation between the National Center for the Exploration of the Oceans of France and the National Council on Marine Resources and Engineering Development of the United States of America,” in: Edward Wenk Jr. to Yves la Prairie, 20 January 1970, ANF, Fonds du Centre National pour l'Exploitation des Océans (1961–1984) (hereinafter CNEXO), 20080658, Box 22. Emphasis mine.

24 COMEXO, “Rapport du Comité d’études ‘Exploitation des Océans,’” 12 March 1960, ADF, RCST, 236QO, Box 35. The DGRST was a scientific office, under the Prime Minister's office, created in 1958 to organize and coordinate special programs in priority scientific and technological fields; Duclert 2004.

25 Pratt 1990. About the transformation of earth sciences after the IGY, including the role played by the IGY in the next UN meetings to draw borders in the seas, see Collis and Dodds 2008.

26 SCOR was a committee established to address interdisciplinary questions related to the oceans under the International Council for Scientific Unions (ICSU), an international body integrating scientific unions.

27 COMEXO, “Rapport du Comité d’études ‘Exploitation des Océans,’” 12 March 1960, ADF, RCST, 236QO, Box 35.

28 About the IACOMS’ creation, aims and selection of representatives, as well as a detail on how it complemented SCOR, see Brunn 1958. On the pursuit of national interests during international cooperation at the IACOMS, see Roberts 2013.

29 Ramsay was an American physicist from Harvard University who, after working on the Manhattan project in 1943, became the first Science Adviser to the NATO Secretary General in 1958.

30 Norman F. Ramsay, “Co‐Operative Oceanographic Research: Note by the Science Advisor,” 30 April 1959, NATO unclassified document, C‐M(59)44, NATO Archives Online, available at: https://archives.nato.int/co‐operative‐oceanographic‐research (accessed 10 October 2020).

31 The creation of this report is detailed in Robinson 2018.

32 A detailed history of the OCR can be found in Turchetti 2012. To situate it in a longer history of NATO's Science Committee, see Turchetti 2019.

33 ORC Minutes of the First Meeting, 12–13 September 1959, NATO unclassified document, AC/137‐D/50, NATO Archives Online, available at: https://archives.nato.int/report‐on‐first‐meeting‐of‐sub‐committee‐on‐oceanographic‐research (accessed 10 October 2020).

34 COMEXO, “Rapport du Comité d’études ‘Exploitation des Océans,’”12 March 1960, ADF, RCST, 236QO, Box 35.

35 Ibid.

36 Besides Lacombe and Eyrès, the COMEXO was constituted by the marine biologists Louis Fage (MNHN), Maurice Fontaine (Director of the Institut Océanographique de Paris), Pierre Drach (University of Paris), Jean‐Marie Pérès (University of Marseille), Jean Furnestin (President of the Conseil International pour l'Exploration de la Mer), the geologist Jacques Bourcart (University of Paris), and two public celebrities: Jacques Cousteau (from the Musée Océanographique de Monaco) and the pioneer of the bathyscaph *FNRS‐2* Théodore Monod.

37 This amount represented 4,1 % of the budget devoted by the DGRST to special actions. All currencies have been converted to 1960’s US dollars.

38 Investing 150,700 dollars for 3 years in NATO projects, and 142,500 dollars for 2 years in UNESCO's. COMEXO, “Contrats de formation en océanographie: rapport pour la période 1965–66,” undated, ANF, Collection: Institut français de recherche pour l'exploitation de la mer (IFREMER). Archives du contrôle sanitaire des établissements et des coquillages (1908‐1972) (hereinafter IFREMER), 20110381, Box 1, Folder “Contrat no. 6600164 avec le Professeur Pères”; COMEXO, “Rapport du Comité d’études ‘Exploitation des Océans,’”12 March 1960, ADF, RCST, 236QO, Box 35.

39 Camprubí 2020.

40 Turchetti 2012.

41 COMEXO, “Contrat N.62‐FR‐07 avec M. Alinat (Institut Océanographique de Monaco) pour l’équipement d'un navire de sondage topographique,” 1962, ANF, IFREMER, 20110381, Box 1; Camprubí and Robinson 2016.

42 Vichney 1962, entitled “Why France is lagging in oceanography.”

43 André Giraud, “Note sur la création d'un etablissement public pour le développement de l'océanographie et la mise en valeur de la mer,” 28 February 1966, ANF, MM CNEXO, 20060160, Box 1; CNEXO 1968.

44 COMEXO, “Rapport d'activité annuel du Comité scientifique ‘Exploitation des Océans,’” 16 March 1967, ANF, MM CNEXO, 20060160, Box 1.

45 Sayle 2019. For an analysis framed in NATO's Science Committee, see Turchetti 2019.

46 Martin 2016.

47 About the interest of President Johnson in promoting oceanography, see: Doel and Harper 2006; Adler 2019, on 101–134 (Chapter: “Cold War Science on the Seafloor”). A detailed history of oceanography under Johnson's Presidency can be found in Hamblin 2005.

48 For a first‐hand account of the creation of the Commission, see: Merrell et al. 2001; Wenk 1968; Wenk 1972; also Scheiber 1998.

49 For a first‐hand account of the US policies and measures to promote ocean exploration, see Wenk 1972.

50 Daniel Frèrejacque, “Eléments d'information pour M. le Président du CNEXO: l'océanographie aux États‐Unis,” 17 July 1967, ANF, Collection: Fonds de la présidence de l′IFREMER et du CNEXO (1967‐2013) (hereinafter Présidence IFREMER), 20160259, Box 328.

51 [Anon.], “Un milliard de francs pour l'océanographie américaine,” undated, ANF, MM CNEXO, 20060160, Box 1.

52 André Giraud, “Rapport sur un projet de création d'un centre national d’études océanographiques,” 25 February 1965, ANF, MM CNEXO, 20060160, Box 1; André Giraud, “Rapport au Comité interministériel de la recherche scientifique et technique,” 29 March 966, ANF, MM CNEXO, 20060160, Box 1.

53 Ibid. (both sources referenced in fn 52).

54 Yves la Prairie to the Minister of Scientific Research and Atomic and Space Affairs, 29 August 1967, ANF, Présidence IFREMER, 20160259, Box 328.

55 La Prairie 1990, on 349.

56 Hamblin 2005, on 141–143 has discussed one more reason to justify why an expert outside the discipline could be selected to drive oceanography. At the US, the harsh rivalry between oceanographic institutions forced the National Academy of Sciences to choose a geochemist, Harrison Brown, to implement a national oceanographic program, rather than one of the directors of the main institutions.

57 His admiration for the General was such that he entitled his memoirs *Ce siècle avait de Gaulle* (This Century had de Gaulle), where he narrates his career in parallel to that of the General; La Prairie 1990. In it, he details his relationships with policy‐makers related to the Gaullist government.

58 Some of these cases have been presented in: Wang 1992; Oreskes and Rainger 2000. In France, the most well‐known case is that of Pierre Joliot‐Curie at the CEA; see Hecht 1998, on 55–60.

59 A detailed history of French submersibles can be found in Jarry 2003.

60 The Soviet Academy of Sciences and the French CNRS had been cooperating since the early 1950s. See Guthleben 2016 for a detailed account of French‐Soviet relations in sciences. De Gaulle's ideas regarding to the USSR are detailed in Martin 2016.

61 About de Gaulle's diplomatic visit to Moscow, see: Lipkin 2016; Martin 2016.

62 Although Franco‐Soviet scientific exchanges were not new, it was the first time they were framed in a cooperative agreement. As Guthleben 2016 has shown, French academic researchers had previous contacts with Soviet institutions through the CNRS. The author argues that the CNRS’ international relations during the Cold War oscillated from one bloc to the other depending on pragmatic reasons: If, after World War II, the French government fought against any reminiscence of communism in the country, during the ‘50s collaborations with eastern countries grew stronger when the communist bloc pioneered in technoscientific advancements, like—as Guthleben exemplifies—the Sputnik's launch or the assemblage of the first intercontinental ballistic missile.

63 Jean Emery, “Note d'information sur les activités scientifiques et techniques du Commandant Cousteau et sur la collaboration franco‐soviétique,” 21 June 1967, p. 5, ANF, Présidence IFREMER, 20160259, Box 328.

64 CNEXO, “État de la coopération franco‐soviétique en matière d'océanographie au 10.12.1968,” 9 [sic!] December 1968, ANF, CNEXO, 20080658, Box 22.

65 Martin 2016; Reyn 2010, on 307–354 (Chapter: “Grand Designs Go Bankrupt: From Divergence to Accommodation, 1967–1969”).

66 About scientists as agents of scientific intelligence, see: Doel 1997; Doel and Needell 1997; Adamson 2016. Robinson 2018, on 113–151 describes the oceanographer George Deacon as a hybrid figure in British oceanography mediating between international collaboration, diplomacy and intelligence gathering.

67 According to la Prairie 1999 (his memoirs), he first contacted with Wenk on 7 April 1967 (p. 353), but there is no written evidence in la Prairie's correspondence until 20 December 1967 where he answers a previous letter from Wenk: Yves la Prairie to Edward Wenk, 20 December 1967, ANF, Présidence IFREMER, 20160259, Box 328. La Prairie highlights Wenk's interest on the CNEXO in both sources.

68 Yves la Prairie to Edward Wenk, 20 December 1967, ANF, Présidence IFREMER, 20160259, Box 328; Yves la Prairie to Jean‐Louis Chaussende (deputy chef to Maurice Schumann, Ministre d'Etat chargé de la Recherche Scientifique), 17 October 1967, Paris, ANF, Présidence IFREMER, 20160259, Box 328. The correspondence at the Archives Nationales de France seems to be incomplete, and does not include most of the incoming letters by Wenk.

69 Yves la Prairie to Daniel Frèrejacque (Scientific Attaché at the French Embassy in the US), 12 September 1967, ANF, Présidence IFREMER, 20160259, Box 328. Several of these reports can be found at the Archives Nationales de France, for instance: Daniel Frèrejacque, “Éléments d'information pour M. le Président du CNEXO: l'océanographie aux États‐Unis,” 17 July 1967, ANF, Présidence IFREMER, 20160259, Box 328; Daniel Frèrejacque, “Nouveau plan d'action en faveur de l'océanologie aux États‐Unis,” 21 January 1969, ANF, CNEXO, 20080658, Box 22.

70 In this case, they were referring to non‐military research, but industries devoted to exploitation of natural resources and technological development. By research centers they were pointing to the Scripps Institution of Oceanography and the Lamont Doherty Earth Observatory.

71 For a contemporaneous account of the LDEO's creation and its director, Maurice Ewing, see Wertenbaker 1974.

72 Yves la Prairie to the Minister of Scientific Research and Atomic and Space Affairs, 29 November 1967, ANF, Présidence IFREMER, 20160259, Box 328; Xavier le Pichon, “Rapport confidentiel: réflexions sur l’état de l'océanographie française et ses possibilités de développement en géologie, géophysique et physique,” 10 October 1968, ANF, Présidence IFREMER, 20160259, Box 327.

73 It included the offshore oil industry and the fishing industry, but also companies devoted to all kind of underwater technologies: from pipes, drillers, and specialized vessels, to submersibles and underwater cabins. For cases of study relating the industry, underwater technologies, and the exploitation of marine resources, see: Rozwadowski and Van Keuren 2004; Adler 2019, on 101–134 (Chapter: “Cold War Science on the Seafloor”). Source: Mission Scientifique (French Embassy in the US), “L'enseignement des disciplines océanographiques aux États‐Unis,” 1 January 1970, ANF, CNEXO, 20080658, Box 22.

74 Yves la Prairie “Formation des chercheurs: note pour le Premier Ministre,” 18 October 1967, ANF, Présidence IFREMER, 20160259, Box 328.

75 Chandra Mukerji has analyzed those roles in American oceanography in Mukerji 1989.

76 Doel 1997.

77 CNEXO, “Contrat 68/43 géothermique et magnétisme marines. Responsables: J. Bonnin et B. Leclerc du Sablon,” 1968 (undated), ANF, IFREMER, 20110381, Box 7. In the report, Bonnin mentions that he acquired and brought back to France the same measurement system to conduct paleomagnetic analysis as the one available at LDEO.

78 Laubier 2002 details the disagreements between the CNEXO and the CNRS. Some geologists have detailed these oppositions and frictions in their memoirs: Boillot 2012; Hommeril 2007.

79 Jean‐François Piccard, Interview with Claude Lalou, 18 June 1986, Archives orales du CNRS, online: http://www.histcnrs.fr/archives‐orales/lalou.html (accessed 19 August 2020). Mukerji 1989 points to the state's capacity to orientate scientific research by controlling vessels and research technologies.

80 Oreskes 2003 has discussed the concept of a *context of motivation* to justify the mutual interest to cooperate between American oceanographers and the US Navy.

81 On the other hand, the number of contracts signed during that period with engineering and fishing companies was 34, a few less than the universities signed.

82 Several examples can be found at the National Archives of France containing the correspondence of the CNEXO's General Director, among those la Prairie's letters to the Minister of Scientific Research and Atomic and Space Affairs reporting him about the discussions held during an international meeting in Brussels to coordinate European cooperation in oceanography (Yves la Prairie to the Minister of Scientific Research and Atomic and Space Affairs, 13 February 1968, ANF, Présidence IFREMER, 20160259, Box 327), or a confidential report presenting envisioned alliances with national and international organisms (Yves la Prairie to the Minister of Scientific Research and Atomic and Space Affairs, 11 November 1967, ANF, Présidence IFREMER, 20160259, Box 328); la Prairie's concerns sent to the Delegate Minister about France's involvement in NATO's scientific campaign MILOC in Norway (Yves la Prairie to the Delegate Minister, 20 June 1969, ANF, Présidence IFREMER, 20160259, Box 326); his advices to the Minister of Foreign Affairs about the proposals to be discussed at the 5^th^ COI meeting (Yves la Prairie to the Minister of Foreign Affairs, 12 September 1967, ANF, Présidence IFREMER, 20160259, Box 328); reports written by the CNEXO's responsible of international relations about UN meetings (Mariani, “Compte rendu de la deuxième session du groupe de travail sur les questions juridiques liées aux recherches scientifiques sur l'océan (New York, 16–20 février 1970),” 13 April 1970, ANF, CNEXO, 20080658, Box 24) and during the CIESM meetings (Alain Sciard, “Réunion du bureau de la CIESM à Monaco, 26 mai 1970,” 5 June 1970, ANF, CNEXO, 20080658, Box 24).

83 A detailed account of the IDOE, as well as previous international ventures of American oceanography, can be found in Jennings 2000.

84 The discourse of cooperation for peace performed by Johnson has been discussed in Hamblin 2005.

85 The Lamont‐Doherty Earth Observatory, the Woods Hole Oceanographic Institution, the University of Miami, and the Scripps Institution of Oceanography; Yves la Prairie to Edward Wenk, 20 December 1967, ANF, Présidence IFREMER, 20160259, Box 328.

86 Yves la Prairie to the Minister of Scientific Research and Atomic and Space Affairs, 20 March 1968, ANF, Présidence IFREMER, 20160259, Box 327.

87 NOAA was established in October 1970 after the recommendations issued by the National Commission on Marine Science, Engineering and Resources.

88 ANF, CNEXO, 20080658, Box 22, Folder “Centres de Recherche 1969.”

89 The COB was built between 1968 and 1971, devoting a 2‐million‐dollar budget; CNEXO, “Fiche sur le futur centre d'océanologie de Brest,” undated, ANF, Présidence IFREMER, 20160259, Box 328. As Robinson 2018 shows, the British had previously done the same: They visited American facilities with the aim of replicating them in the UK during the 1940s.

90 Hardy 1969.

91 By 1967 half of the French oceanographers worked in Parisian universities, whereas there were twice as many Mediterranean research centers as on the Atlantic coast; Yves la Prairie to the Minister of Scientific Research and Atomic and Space Affairs, 29 November 1967, ANF, Présidence IFREMER, 20160259, Box 328.

92 Hardy 1969.

93 See Reyn 2010, 307–354 (Chapter: “Grand Designs Go Bankrupt: From Divergence to Accommodation, 1967–1969”).

94 Carmoy 1969; Reyn 2010, on 334–354.

95 Mission Scientifique, “Evolution de la coopération scientifique et technique franco‐américain,” ANF, CNEXO, 20080658, Box 22.

96 Daniel Frèrejacque, “Nouveau plan d'action en faveur de l'océanologie aux États‐Unis,” 21 January 1969, ANF, CNEXO, 20080658, Box 22; Mission Scientifique (French Embassy in the US), “L'enseignement des disciplines océanographiques aux États‐Unis,” 1 January 1970, ANF, CNEXO, 20080658, Box 22.

97 CNEXO 1970.

98 According to a letter from Yves la Prairie to the Minister of Scientific Research and Atomic and Space Affairs, 20 March 1968, ANF, Présidence IFREMER, 20160259, Box 327

99 Edward Wenk Jr., “Terms of Reference for Marine Science Cooperation between the National Center for the Exploitation of the Oceans of France and the National Council on Marine Resources and Engineering Development of the United States of America,” in: Edward Wenk Jr. to Yves la Prairie, 20 January 1970, ANF, CNEXO, 20080658, Box 22

100 Hamblin 2005, on 244.

101 Jacques Perrot, Conference “Aspects économiques de l'exploitation des océans” at the Association des Officiers de Réserve de la Marine Nationale (ACORAM) in April 1970, undated, p. 8, Paris, ANF, Présidence IFREMER, 20160259, Box 323.

102 Robinson (forthcoming) analyzes the same will but from the American perspective, where policy‐makers and oceanographers interested in marine exploitation were eager to be the ones who would “conquer the oceans” first.

103 Naomi Oreskes has presented the project FAMOUS from the American's perspective in Oreskes 2003. The project's organization from the US has been described in Ballard 2000.

104 Translated from the original: “[J'ai été] un lien vivant et permanent entre la politique et la science: Interprète du savant pour le monde politique. Interprète du politique pour le monde de la science.” La Prairie 1990, on 405, while reflecting on the role he had played during his career at the CEA and at the CNEXO.
